# Supplementary material for: Dual-feature selectivity enables bidirectional coding in visual cortical neurons
Source: eLife. 2026 Nov 9;15:RP109861. doi: 10.7554/eLife.109861 (PMC13747320; doi:10.7554/eLife.109861)
Supplement: Peer review history

# Editor report: eLife Assessment

**DOI:** 10.7554/eLife.109861.3.sa0

**Authors:** Tirin Moore

**Affiliations:** Stanford University, Howard Hughes Medical Institute United States

**Keywords:** Compelling, Fundamental

The authors combine a modeling approach, using a digital twin, with electrophysiological evidence in two species to assess the role of inhibition in shaping selectivity in the visual cortex. The results provide a **fundamental** advance beyond the classic view of sensory coding by proving **compelling** evidence that many neurons in visual areas exhibit dual-feature selectivity. Overall, the work compellingly showcases how in silico experiments can generate concrete hypotheses about neuronal coding that are difficult to discover experimentally.

# Referee report: Reviewer #1 (Public review)

**DOI:** 10.7554/eLife.109861.3.sa1

The multi-species approach of testing the model in macaque and mouse is excellent, as it improves the chances that the observed findings are a general property of mammalian visual cortex. It would be useful to delineate however any notable differences between these species, which are to be expected given their lifestyle.

The overall performance of the model appears to be excellent in V1, with over 80% performance, but falls substantially in V4. It would be important to consider the implications of this finding; for example, in the context of studying temporal lobe structures that are central to recognizing objects. Would one expect that model performance decreases further here, and what measures could be taken to avoid this? Or is this type of model better restricted to V1 or even LGN?

While the manuscript delineates novel axes of inhibitory interactions, it remains unclear what exactly these axes are and how they arise. What are the steps that need to be taken to make progress along these lines?

Comments on revised version.

The authors have adequately addressed the points I raised in my review during the revision.

# Author comment: Author response

**DOI:** 10.7554/eLife.109861.3.sa2

**Authors:** Nikos Karantzas, Katrin Franke, Konstantin Friedrich Willeke, Maria Diamantaki, Kandan Ramakrishnan, Hasan Atakan Bedel, Pavithra Elumalai, Kelli Restivo, Paul Fahey, Cate Nealley, Tori Shinn, Gabby Garcia, Saumil Patel, Alexander Ecker, Edgar Walker, Emmanouil Froudarakis, Sophia Sanborn, Fabian H Sinz, Andreas Savas Tolias

**Affiliations:** Stanford University Stanford United States; Stanford University Stanford United States; University of Tuebingen Tuebingen Germany; Foundation for Research and Technology-Hellas | Institute of Molecular Biology and Biotechnology, Greece Heraklion, Crete Greece; Baylor College of Medicine Houston United States; Stanford University Stanford United States; University of Göttingen Göttingen Germany; Baylor College of Medicine Texas United States; Stanford University Stanford United States; Stanford University School of Medicine Stanford United States; Baylor College of Medicine Texas United States; Stanford University Stanford United States; Stanford University Stanford United States; University of Göttingen Lower Saxony Germany; University of Washington School of Medicine Seattle United States; University of Crete Heraklion Greece; Stanford University Stanford United States; University of Göttingen Göttingen Germany; Stanford Medicine Stanford United States

The following is the authors’ response to the original reviews.

**Public Reviews:**

**Reviewer #1 (Public review):**

This manuscript used deep learning to highlight the role of inhibition in shaping selectivity in primary and higher visual cortex. The findings hint at hitherto unknown axes of structured inhibition operating in cortical networks with a potentially key role in object recognition.

The multi-species approach of testing the model in macaque and mouse is excellent, as it improves the chances that the observed findings are a general property of mammalian visual cortex. However, it would be useful to delineate any notable differences between these species, which are to be expected given their lifestyle.

The overall performance of the model appears to be excellent in V1, with over 80% performance, but it falls substantially in V4. It would be important to consider the implications of this finding; for example, in the context of studying temporal lobe structures that are central to recognizing objects. Would one expect that model performance decreases further here, and what measures could be taken to avoid this? Or is this type of model better restricted to V1 or even LGN?

While the manuscript delineates novel axes of inhibitory interactions, it remains unclear what exactly these axes are and how they arise. What are the steps that need to be taken to make progress along these lines?

**Reviewer #2 (Public review):**

The classic view of sensory coding states that (excitatory) neurons are active to some preferred stimuli and otherwise silent. In contrast, inhibitory neurons are considered broadly tuned. Due to the gigantic potential image space, it is hard to comprehensively map the tuning of individual neurons. In this tour de force study, Franke et al. combine electrophysiological recordings in macaque (V1, V4) and mouse (V1, LM, LI) visual cortex with large-scale screens based on digital twin models, as well as beautiful systems identification (most/least activating stimuli). Based on these digital twins, they discover dual-feature selectivity (which they validate both in macaques and mice). Dual-feature selectivity involves a bidirectional modulation of firing rates around an elevated baseline. Neurons are excited by specific preferred features and systematically suppressed by distinct, non-preferred features. This tuning was identified by excellently combining advances in AI & high-throughput ephys.

The study is comprehensive and convincing. Overall, this work showcases how in silico experiments can generate concrete hypotheses about neuronal coding that are difficult to discover experimentally, but that can be experimentally validated! I think this work is of substantial interest to the neuroscience community. I'm sure it will motivate many future experimental and computational studies. In particular, it will be of great interest to understand when and how the brain leverages dual-feature selectivity. The discussion of the article is already an interesting starting point for these considerations.

Strengths:

(1) Using computational models to predict neuronal responses allowed them to go through millions of images, which may not be possible in vivo.

(2) The cross-species and cross-area consistency of the results is another major strength. Pointing out that the results may be a fundamental strategy of mammalian cortical processing.

(3) They show that the feature causing peak excitation in one neuron often drives suppression in another. This may be an efficient coding scheme where the population covers the visual manifold. I'd like to understand better why the authors believe that this shows that there are low-dimensional subspaces based on preferred and non-preferred stimulus features (vs. many more, but some axes are stronger).

We thank the reviewers for their constructive and helpful feedback on our manuscript. We are delighted that they found the study to be “comprehensive and convincing” and a “tour de force” in its combination of electrophysiological recordings with large-scale digital twin screening. We appreciate that the reviewers highlighted the strengths of our multi-species approach and the “cross-species and cross-area consistency” of the results, noting that the work showcases how *in silico* experiments can generate concrete, experimentally validatable hypotheses. Overall, we agree with the assessment of the reviewers. We have performed the following changes to the text to clarify and strengthen the manuscript, without introducing new analyses or altering the conclusions.

**Recommendations for the authors:**

**Reviewer #1 (Recommendations for the authors):**

(1) Page 3: The authors state that RFs were mapped using sparse noise, with the goal to ensure that the RFs align with the visual stimulus, but no data appear to be shown regarding this alignment. It would be important to provide a full analysis of the sparse noise-mapped RFs for both V1 and V4. Also, is it correct that the V4 data analyzed here came from a single animal? This could potentially be problematic and would need to be addressed, for example, by performing analyses also in V1 for participant animals separately. Please elaborate.

We have added a sentence to the Results section clarifying the sparse noise RF mapping procedure, noting that probe insertions were targeted orthogonal to the cortical surface so that neurons sampled along the probe depth share overlapping receptive fields, allowing a single stimulus configuration to adequately drive the entire recorded population. We have also corrected the text to clarify that V4 data were collected from 2 animals (not 3 as previously stated in an earlier draft), consistent with the Methods section.

(2) Page 4: Only half the neurons in V4 are "high confidence" in terms of test image performance, which seems a little low and probably significantly lower than the corresponding value for V1 of 84%. It is unclear how to interpret this confidence, but it seems to suggest that half of the V4 neurons are not well captured by the model. If true, this fraction appears large enough to cast doubt on the validity of the V4 results. Please elaborate.

We have expanded the text to explicitly discuss the lower proportion of high-confidence *in-silico* neurons in V4 relative to V1. We attribute this to the greater complexity of V4 tuning compared to V1, as well as missing contextual information such as image surrounds and sequential image context—factors that likely limit model performance in higher visual areas. We note that our restriction of analyses to high-confidence neurons provides resilience against these limitations, and that the goal was not to maximize predictive performance per se but to identify response patterns—dual-feature selectivity—that are robust across neurons, areas, and species.

(3) Page 5: It seems that identical L2 norms are valid for discounting contrast variations, particularly if the neural responses are linear, since the L2 norm is computed on the entire RF. It might be judicious to attenuate the claim that contrast variation has no effect.

We have softened the claim that contrast variation has no effect. The revised text now states that L2 normalization controls for root-mean-squared contrast but does not fully equate effective contrast in nonlinear cells, whose responses depend on the spatial structure of the stimulus beyond its total energy. We note that residual contrast dependent effects, particularly in the suppressive regime, cannot be entirely excluded.

(4) Page 6: The authors acknowledge that, at least for simple cells, a phase shift in the grating and concomitant ON-OFF overlap is an inhibitory axis, which is correct. It does not really become clear what other axes were found, and whether any of these represent a novel discovery about V1.

We have clarified the description of inhibitory axes in V1, noting that while phase-shifted stimuli represent a well-established suppressive axis for simple cells reflecting linear On-Off subfield structure, and complex cells exhibit no coherent suppressive pattern due to phase pooling, neither model class accounts for the multidimensional suppressive structure we observe. We have made explicit that our unbiased approach reveals suppressive structure spanning simultaneous changes across orientation, spatial frequency, phase, and texture, exceeding what any single known suppressive mechanism predicts.

(5) Page 7: Dreamsim is based on human similarity judgements, whereas the data is from macaques. Is there any evidence suggesting that macaque similarity judgements might be similar to those of humans?

We have added a paragraph to the Discussion acknowledging that DreamSim was trained on human perceptual similarity judgments while our neuronal data are from macaques. We note that this cross-species application is supported by the deep homology between primate ventral visual streams, and that natural-image similarity judgments have been found to be highly consistent across macaques and humans. Importantly, we clarify that we deploy DreamSim not as a model of macaque perception but as an image feature embedding to test whether stimuli that cluster in perceptual space evoke similar neuronal responses—a use that is robust to the precise calibration of the metric. We also note that we are developing custom macaque-specific embeddings for future work.

(6) Page 7: How many images were in the test set?

We have added the number of test images to the relevant text (*n*=75 for V1, *n*=150 for V4) and to the Figure 1 caption.

(7) Page 8: As mentioned above, performing the analysis on V1 data of individual subjects and demonstrating similar digital twins might be an additional way to confirm the models' accuracy.

We have added text noting that for V4, 1digital twin models were fit independently per neuron without sharing information across animals, and that extreme image sets identified by the model elicited correspondingly extreme responses in neurons from the other animal, confirming that identified selectivity patterns are not idiosyncratic to individual subjects.

(8) Page 11: The mouse data is presented very briefly only, and the authors seem to imply that there is a high degree of coding similarity between this rodent species and macaques and, by extension, humans. Were there any notable differences between the mouse and macaque data?

We have added text explicitly noting that while macaque and mouse visual cortex differ substantially in their functional organization and the complexity of neuronal selectivity, the broader principle—that non-sparse neurons are jointly defined by distinct excitatory and suppressive feature sets—generalizes across mammalian visual systems. We clarify that this does not imply that mouse and macaque visual cortex share similar functional organization or equivalent complexity of neuronal selectivity; rather, within the representational regime of each area, neurons are organized such that excitatory and suppressive feature sets are jointly structured and distinct.

(9) Page 13: One main finding of the study is that inhibition appears to operate along additional dimensions that had not been previously recognized, but what is the nature of these dimensions, how do they arise and relate to known inhibitory effects in V1 such as centre-surround effects? The fact that suppression is tuned in response to natural images or other complex objects is not a new finding, and there is plenty of published work along these lines; the authors may want to cite Tamura et al 10.1152/jn.01267.2003. I am not sure introducing the term "dual feature selectivity" is really a major conceptual advance.

We have added a citation to Tamura et al. (2004) in the Discussion, alongside other prior work documenting suppression by non-optimal stimuli. We have also expanded the Discussion to more carefully position our findings relative to existing work on feature-selective suppression, noting that while prior work has established that inhibition can be structured and feature-selective, our results suggest a broader organizing principle: within each visual area, there exists a set of feature combinations from which individual neurons draw both their excitatory and suppressive preferences.

(10) Page 14: The authors enumerate a number of technical limitations, which is to be commended. It would be useful for them to comment on the particular advantages of the digital twin model, compared to a more traditional analysis of the responses to the thousands of natural images that were experimentally obtained. It seems likely that the main finding, i.e. tuned inhibition, is also evident directly in this population (?). While the digital twin is to some degree validated by the test images, its responses to the much larger set of images studied are not validated, and one must trust that the ResNet50 indeed captures V4 selectivity. It would be useful to discuss some of these points, and highlight a potential way that digital twins (maybe as a shared model between laboratories) can learn from a large number of animals and datasets, and maybe even be used to generate novel visual stimuli suitable to test emergent hypotheses.

We have added a paragraph to the Discussion explicitly contrasting the advantages of digital twin models with direct analysis of experimentally recorded responses, noting that digital twins enable screening of more than one million images per neuron *in silico*, gradient-based synthesis of stimuli precisely optimized to drive or suppress individual neurons, and cross-model verification of identified selectivity patterns—a test that has no analog when working with fixed experimental image sets.

**Reviewer #2 (Recommendations for the authors):**

Minor comments:

(1) Call out Figure 1/b in the main text.

We have added a callout to Figure 1b in the main text

(2) Can you make a supplementary figure illustrating more examples with skewness around the middle (e.g. 1.5, 2, 2.5)? Namely, you state that 2 is a good threshold for deciding if it is non-sparse, but you only present clear-cut cases in Figure 2 (with <0.75 and >3.5). I am wondering if 2 is a good threshold?

We have revised the text to clarify that the skewness threshold of 2.0 is adopted purely for analytical convenience to focus subsequent analyses on neurons with sufficiently graded response distributions, and that the key findings are not dependent on the exact threshold chosen. We explicitly note that the underlying distribution of sparsity is continuous, consistent with recent findings (Gondur et al., 2025).

(3) The reference "A tale of two tails: Preferred and anti-preferred natural stimuli in visual cortex." Has no authors. I know it's anonymous, but maybe put that for now? I also congratulate including a paper that is anonymously under review at ICLR 2026. I don't find Unk, 2025 in the list of references. Perhaps related?

We have updated the reference “A tale of two tails” to include the authors (Gondur et al., 2025) and ensured it appears consistently in the reference list. We have also resolved the missing “Unk, 2025” citation, which now correctly refers to this same work.

(4) Why do you use a different model for the analysis in Figure 8?

We have added text to the Methods and Results clarifying why a distinct architecture was used for the V4 evaluator model in Figure 8. Specifically, the V4 generator model uses a fixed, pretrained ResNet50 backbone whose weights are deterministic; any re-trained model sharing this backbone would not constitute a genuinely independent evaluation. By contrast, for V1, the ConvNeXt core is fine-tuned from different random initializations, producing architecturally equivalent but computationally independent models. A truly independent V4 evaluator therefore required a fundamentally different architecture.
